# Supplementary material for: Are carbon emissions trading and green financial instruments synergistic? -Comprehensive quantitative research based on content analysis
Source: PLoS One. 2024 Mar 7;19(3):e0298601. doi: 10.1371/journal.pone.0298601 (PMC10919601; doi:10.1371/journal.pone.0298601)
Supplement: S1 Data — (ZIP) [file pone.0298601.s001.zip › Data/地级市Z_F工作报告.zip!/┴╔─■/┴╔╤⌠╩╨/└┤╫╘╣1⁄2╓┌║┼í╛┬φ┐╦╩2╛▌═°í┐_2016.docx]

各位代表：

　　我代表市政府向大会报告工作，请予审议，并请市政协委员和列席会议的同志提出意见。

　　一、“十二五”时期的主要成就和2015年工作回顾

　　过去的五年，是我市发展进程中极不平凡的五年。五年来，在省委、省政府和市委的领导下，市政府团结带领广大干部群众，坚持稳中求进工作总基调，积极应对经济下行压力，奋力拼搏，开拓进取，“十二五”规划实施取得积极成效，主要目标任务基本完成。预计地区生产总值年均增长8.5%，固定资产投资年均增长6%，社会消费品零售总额年均增长13.3%，外贸出口年均增长15%。

　　产业结构调整步伐加快。三次产业结构由2010年的6.2：63.3：30.5调整到7∶55.7∶37.3。农业基础地位进一步巩固，综合生产能力持续提升。工业结构不断优化升级，芳烃及精细化工、铝合金精深加工、装备制造及汽车零部件产业向高端化、集群化迈进。特色服务业集聚效应凸显，金融、旅游、物流等各类业态齐头并进，服务业占比提升6.8个百分点。

　　改革开放实现新突破。全面完成新一轮政府机构改革。大力实施简政放权，取消和下放市级行政职权437项。市属国有企业改革进一步深化，国有资产监管体制不断完善。商事、财税、医药卫生等领域改革扎实推进。信用辽阳建设全面启动。对外合作广度深度逐步拓展，全方位对外开放格局基本形成。

　　城乡面貌发生深刻变化。河东新城起步区城市形象初步树立，新老城区实现跨河而立。老城区改建加速推进，城市建成区面积扩大到103平方公里。积极构建“一区两轴两翼”的新型城镇化格局，常住人口城镇化率达到62%。生态文明建设取得成效，“青山、碧水、蓝天”工程扎实推进，节能减排等约束性指标全面完成。

　　社会各项事业蓬勃发展。各级各类教育全面加强，城区基本实现义务教育均衡发展。建立健全了市、县、乡三级政府公共卫生管理和服务机构，人均卫生资源占有量居全省前列。文化体育惠民工程扎实推进，图书馆、健身大厦等文体设施相继落成。连续七次荣获全国双拥模范城称号。社会和谐稳定局面不断巩固。

　　人民生活水平显著提高。累计新增城镇实名制就业24.7万人，实现了创建充分就业城市的目标。覆盖城乡的社会保障体系不断完善，保障水平逐年提高。保障性住房建设有序推进。城乡常住居民人均可支配收入年均分别增长11.8%和12.7%。

　　“十二五”时期，全市经济社会发展取得的成绩，为新一轮全面振兴奠定了坚实基础。

　　各位代表，刚刚过去的2015年，全市经济发展遇到了一系列新问题新挑战，多重困难和矛盾相互交织，各种风险和压力比预想的要大。在挑战面前，我们主动作为，迎难而上，保持了经济社会健康平稳发展。预计地区生产总值实现 1025亿元，一般公共预算收入实现67亿元，固定资产投资实现500亿元，城乡常住居民人均可支配收入分别实现26330元和12100元。

　　一年来，我们重点抓了以下八项工作。

　　（一）全力以赴稳增长

　　把稳增长作为全市经济工作的核心任务，确保经济运行在合理区间。

　　千方百计稳企业。开展“千名领导干部包扶千户企业”活动，帮助企业解决困难和问题560余项。安排“过桥”资金3亿元、中小微企业助保贷风险补偿金1.2亿元，努力破解企业转贷难和融资难问题。金融机构各项贷款余额突破千亿元，创历史新高。召开民营经济发展大会，出台《加快中小微企业发展的若干意见》，推进大众创业、万众创新，新增内资企业3035户，增长13.3%。

　　着力抓有效投资。坚持重大项目调度制度，扎实推进225个投资亿元以上项目建设，促成23个重点工业项目竣工投产。积极推进政府和社会资本合作，设立30亿元启动引导金，污水处理厂PPP项目实现引资5.4亿元，北环路等交通基础设施项目取得实质性进展。深入落实国务院28号文件精神，扎实推进“四个一批”项目，东辽阳老工业区搬迁改造项目纳入国家支持范围，弓长岭独立工矿区改造搬迁工程获得国家支持。相继赴欧洲、京津冀、长三角、珠三角等地开展招商活动，跟踪推进央企沪企辽宁行签约项目，促成施耐德汽车零配件等13个项目落地。

　　积极促进消费提档升级。大力推进六大领域消费，培育了信息、文化体育等消费新热点。狠抓住房消费，制定并实施促进房地产业健康发展和棚户区改造的政策措施，推进分散化、货币化、市场化安置，举办春秋两季房交会，盘活了大批存量房源。组织大型商场和超市延时营业，活跃“夜经济”。

　　扩大出口规模。积极争取专项扶持资金，切实加强化工、皮装裘皮、农产品等出口基地建设，外贸出口实现7.05亿美元，增长6.3%。深入实施“走出去”战略，引导3户企业实施境外建厂，帮助11户企业将产品打入欧美市场。

　　（二）加速工业结构优化升级

　　大力推进工业结构调整。俄油加工优化增效改造项目可研已获批复，编制并实施了芳烃及精细化工产业基地规划，法液空制氢、华峰苯深加工等系列项目相继竣工投产，芳烃基地向特色化、高端化迈进。实施了200万吨铝板带箔、8万台份铝合金专用车厢等项目建设，千亿元工业铝材精深加工产业集群已具雏形。支持金兴内饰等企业加强技术改造，高端装备制造业初现端倪。培育发展了盾构机、铝合金航空材料等战略性新兴产业。设立了5亿元新兴产业引导基金。加快传统产业转型升级，钢铁产能和结构调整项目通过国家认定。编制了《中国制造2025辽阳行动纲要》，启动了智能制造等八项重点工程。

　　深入实施创新驱动战略。与50多家科研院校建立了紧密型合作关系，促成产学研合作项目30余项。全面实施促进科技成果转化“提质增效”行动，170多项成果列入省项目库。忠旺集团荣获第17届中国专利奖，三三工业公司获批国家地方联合工程研究中心，奥克集团成为国内聚乙二醇型药用辅料最大供应商。完成企业并购3户，引进海外技术1项。

　　加大环境保护力度。全面贯彻落实新环保法，深入开展大气污染整治七个专项行动，拆除城区燃煤锅炉35台，淘汰小钢铁企业11户，购置新能源公交车120台。加强辽河流域水污染治理，双河及周边地区矿山生态修复示范工程被省政府列为“十三五”重点工程。万元地区生产总值综合能耗下降4%。

　　（三）全面提高农业农村发展水平

　　启动国家现代农业示范区建设。实施了首山农业嘉年华等重大项目建设，初步构建了以5个示范区（带）为主体的现代农业发展格局。农业主导产业长足发展。粮食总产量突破100万吨。大力推进永久绿色水稻和优质玉米示范区建设，建成一批现代设施农业小区，实施亿元以上农业产业化项目13个。现代农业保障体系进一步健全。加强集成农业技术推广基地建设。连续13年无区域性重大动物疫情发生。农产品质量追溯体系初步建成。加快推进农业机械化，辽阳县被农业部确定为全国水稻机械化生产示范县。农村气象防御体系实现全覆盖。

　　积极推动农业农村改革创新。深入开展农村土地承包经营权确权登记颁证试点工作，加速推进农业规模化经营，流转土地15.6万亩，新发展农民专业合作社280个、家庭农场210家。创新农业农村金融平台，完善“种粮乐”担保政策，开发“政银保”金融产品，全年为农民发放贷款2.3亿元。

　　加强农业农村基础设施建设。统筹涉农资金建设高标准农田，全省示范试点项目落户我市。新建农村安全水源48处，解决了10万人的饮水安全问题。刘二堡“千吨万人”集中供水工程被确定为全省样板。巩固青山工程成果，强化封山育林，大力治理闭坑矿山。加强农村环境整治，弓长岭区在全省率先实现美丽乡村示范村全覆盖。

　　深入推进精准式扶贫开发。精确识别贫困人口26492人，实施定点扶贫项目200多个，发放扶贫贷款7000多万元，33850名贫困人口实现稳定脱贫。

　　（四）大力发展现代服务业

　　改造提升传统服务业。开工建设了襄平峰汇、万嘉国际生活广场、中南购物中心等大型商业综合体。出台《促进电子商务发展的意见》，引导传统商业与电子商务融合发展，电子商务产业园投入运营，入驻企业32家；辽联集团获评省级电子商务示范企业。

　　加快发展生产性服务业。完成29户工业企业分立生产性服务业工作。宏伟区生产性服务业集聚区晋升为省级集聚区。引进盛京银行等4家外埠金融机构落户我市。引导物流业向新兴领域拓展，建成佟二堡保税仓库，与国际四大拍卖行实现了网上直播竞拍。佟二堡跻身省级新型专业市场示范区行列。我市被省政府确定为农产品现代流通体系建设试点市。

　　大力发展生活性服务业。养老服务中心养员楼、老年宫改造项目竣工。做大做强温泉旅游业，假日热带雨霖馆等项目开工建设，弓长岭区荣获“中国最佳温泉旅游目的地”称号。深入挖掘历史文化旅游资源，太子河历史文化风光带获批国家4A级景区，启动了冮官屯窑址和辽白瓷生产工艺恢复工作。大力发展乡村沟域旅游，深度开发农家特色、农家产品，吸引了省内外大批游客。

　　（五）切实加强城市建设和管理

　　稳步推进河东新城建设。万达城市广场和水上乐园项目主体封顶，中南新城商业综合体建设进展顺利。加快发展便民服务业，商业网点已近500家。水系景观建设扎实推进。襄平大桥竣工，河西堤顶路通车，北部路网不断完善。融资工作取得新进展，第三期城投债和第二批PPN债近期有望获批。

　　加速改建老城区。募集资金64亿元，实施棚改项目30个，惠及居民1.7万余户。综合改造老旧小区20个，维修“弃管楼”230栋。建成望京园，改造了泰和园、新华园和曙光路生态步行街。维修城市主次干道10条、街巷道路36条。燃气、供水、供热等基础设施建设不断加强，环卫机械化作业水平进一步提升。实施了繁荣路、振兴路和南环街升级改造及绿化工程。基本完成站前大型人防交通枢纽前期工作。我市被交通部列为绿色交通低碳环保综合示范市。

　　加强城市管理。启动了《城市总体规划》修改工作。调整和完善城市管理职责，建立健全网格化管理体系，启动数字化指挥中心建设，初步构建了管理与执法统一的“大城管”格局。

　　（六）着力深化重点领域改革

　　积极推进政府职能转变。调整行政审批事项320项，市本级行政审批事项削减到247项。规范行政权力运行，建立了权责清单制度。出台《进一步加强政府投资管理的实施意见》，促进政府投资项目规范化管理。优化建设工程项目审批流程，实行“一个窗口”受理审批。完成了市直事业单位分类改革，启动了机关事业单位工资制度改革。

　　市属国有企业改革不断深化，2户企业完成国有股退出。实施经营性国有资产统一监管改革，完成了33户企业移交工作。扎实做好国有企业“三供一业”分离工作。大力推进企业“退二进三”，一批存量资产得到盘活。商事制度改革全面实施。医药卫生体制改革取得突破性进展，完成两县（市）中心医院综合改革，政府举办的乡镇卫生院或卫生计生服务中心实现全覆盖。

　　（七）扎实推进社会建设

　　深入贯彻中央“坚守底线、突出重点、完善制度、引导预期”的思路和要求，集中全市65%以上的财力用于民生建设。市政府确定的18项民生实事全面完成。

　　大力开展就业援助，新增城镇实名制就业3.5万人，城镇登记失业率控制在3%以内，零就业家庭保持动态为零。完善社会保障体系，新增参保人员3.2万人。进一步提高保障标准，退休人员养老金月均提高201元，城镇居民基本医疗保险政府补助标准人均提高60元。分配公租房1500套，新建公租房621套，发放租赁补贴6200户。改造农村危房606户。

　　全面发展各项社会事业。义务教育均衡发展，高中教育、职业教育质量稳步提高。对25所农村义务教育薄弱学校进行了改造，新改扩建农村幼儿园8所，完成市五中、七中校园改造工程，对市直学校全面实施了消防设施改造。医疗卫生服务体系建设得到加强，市三院儿童病房楼、市二院病房楼投入使用。公共卫生项目全面落实。文化惠民和全民健身活动深入开展，群众性文体活动丰富多彩。国防教育和国防动员建设进一步加强，创建全国文明城市步伐加快。国家安全、民族宗教、外事侨务、涉台事务、海关、检验检疫、防震减灾、人防、人口和计划生育、妇女儿童、物价、地方志、统计、档案、住房公积金、慈善和红十字等事业都取得新进步。

　　加强社会管理综合治理。创新完善立体化社会治安防控体系，多发性案件和公共安全事故得到有效防范。以抗战胜利70周年等重大安保为重点，全面落实“打防管控服”各项措施，确保了社会大局平稳有序。深入开展信访法治建设年和信访积案百日攻坚行动，集中化解了一批信访积案。落实安全生产责任制，遏制了重特大事故发生。健全食品药品监管机制，保障了群众饮食用药安全。创新社会治理，白塔区被确定为第一批全国社区管理和服务创新示范区。

　　（八）进一步加强民主法治建设

　　主动接受市人大及其常委会的法律监督、工作监督，认真执行市人大及其常委会的决议和决定；坚持重大事项决策前向人大报告，积极回应人大代表对社会热点问题的关注，办理人大代表议案1件、建议208件。自觉接受市政协的民主监督，积极与政协开展民主协商，办理政协提案294件。认真听取各民主党派、工商联、无党派人士、人民团体的意见和建议，积极支持工会、共青团、妇联等人民团体依照各自章程开展工作。

　　坚持以上率下，扎实开展“三严三实”专题教育，制定了《关于全面落实党建工作和党风廉政建设责任制的规定》，修订完善了政府工作规则，政府部门工作作风明显好转，机关服务意识和行政效率进一步提高，政风行风建设显著加强。

　　出台《加快推进法治政府建设实施意见》，严格执行重大行政决策合法性审查制度，依法行政水平进一步提高。圆满完成“六五”普法任务，法律服务和法律援助能力不断增强。

　　深入落实党风廉政建设责任制，加大行政监察和审计监督力度，全面加强以“五大系统”为重点的惩防体系建设。健全完善绩效考核体制机制，严格奖惩问责，政府机关及工作人员干事创业的积极性显著提高。

　　各位代表，在经济形势严峻复杂的局面下，取得上述成绩极为不易。这些成绩的取得，是全市人民团结奋斗的结果；是市人大法律监督、工作监督和市政协民主监督、参政议政的结果；是驻军部队，中央、省驻我市单位，各民主党派、人民团体、社会各界人士、外来投资者和建设者关心支持的结果。在此，我代表市政府，向支持参与辽阳经济社会建设的全市人民和各界人士表示衷心的感谢！

　　在总结成绩的同时，我们也清醒地看到经济社会发展和工作中存在的问题和不足。一是经济下行压力较大，部分行业和企业生产经营困难。受宏观经济环境和做实经济数据等多重因素影响，部分经济指标回落较大。二是产业结构偏重，服务业发展相对滞后。创新能力不强，战略性新兴产业发展步伐不快。三是历史形成的体制机制矛盾尚未根本解决，政府性债务、养老金发放、社会风险管控等领域仍然存在一定隐患。四是政府自身建设还有薄弱环节，发展环境有待进一步优化。部分干部有畏难情绪，机关作风、服务水平还没有适应经济发展新常态。对于这些问题，我们要采取有力措施，认真加以解决。

　　二、“十三五”时期的发展战略和2016年的重点工作

　　“十三五”时期，是全面建成小康社会决胜期，是推动辽阳新一轮全面振兴关键期，也是我们可以大有作为的重要战略机遇期。尽管当前经济形势依然严峻复杂，但我市经过多年的振兴发展，打下了较为雄厚的基础，积蓄了很大的发展潜力。新一轮东北振兴和“一带一路”建设等国家战略同步实施，为我们提供了重大历史机遇。特别是建设“四个辽阳”的战略鼓舞人心、催人奋进。在这一战略引领下，全市一心一意谋发展，凝心聚力干事业的氛围更加浓厚，为我们加快发展提供了充足的内生动力。我们要认清发展大势，坚定必胜信心，积极推动“十三五”时期经济社会发展，为辽阳老工业基地全面振兴打下坚实基础。

　　全市经济社会发展的指导思想是：高举中国特色社会主义伟大旗帜，全面贯彻党的十八大和十八届三中、四中、五中全会精神，以马克思列宁主义、毛泽东思想、邓小平理论、“三个代表”重要思想、科学发展观为指导，深入贯彻习近平总书记系列重要讲话精神，按照“四个全面”战略布局要求，牢固树立创新、协调、绿色、开放、共享发展理念，坚持以“四个着力”为根本遵循，适应把握引领经济发展新常态，培育壮大新的经济增长点，保持战略定力，坚持稳中求进，走出一条质量更高、效益更好、结构更优、环境更佳、活力更强的发展新路，确保如期全面建成小康社会，加快建设智慧文明美丽幸福新辽阳，谱写振兴发展新篇章。

　　全市经济社会发展的战略定位和主要任务是：

　　经济保持中高速增长。主要经济指标年均增速高于全省平均水平，经济总量和发展水平在全省位次前移。到2020年，地区生产总值和城乡常住居民人均可支配收入比2010年翻一番。

　　经济发展方式实现重大转变。工业化、信息化深度融合，产业迈向中高端水平，建成具有核心竞争力的三大工业产业基地。农业主导产业转型升级步伐加快，率先基本实现农业现代化。现代服务业加速发展，服务业增加值占比稳步提升。消费对经济增长的贡献不断加大。城镇化质量明显提高，常住人口城镇化率达到66%。

　　创新驱动发展能力大幅提升。科技研发经费投入占地区生产总值比重达到2.5%，科技进步对经济增长贡献率达到60%。全民受教育程度和创新人才培养水平明显提高。以企业为主体的技术创新体系初步形成，自主创新能力全面提升。

　　改革开放实现新突破。重点领域和关键环节改革取得重大成果，全面完成223项改革任务，形成系统完备、科学规范、运行有效的体制机制。开放型经济水平大幅提高，“引进来”与“走出去”协调推进，对外贸易加速转型升级。

　　文化和社会建设明显加强。社会主义核心价值观根植人心，公民文明素质和社会文明程度明显提高。文化产品更加丰富，公共文化服务体系基本建成，文化产业成为国民经济支柱产业。社会治理制度不断健全，法治辽阳建设全面推进。

　　生态环境建设成效显著。主体功能区布局基本形成，耕地保有量保持在265万亩。万元地区生产总值用水量年均下降4.8%，单位地区生产总值能源消耗年均下降3%。森林覆盖率达到39.6%，森林蓄积量达到500万立方米。

　　人民生活质量和水平显著提高。城乡居民人均收入保持与经济发展同步增长，收入分配差距缩小。社会保障体系不断完善，逐步实现基本公共服务均等化、基本权益均等化、要素分配均等化、收入均衡化，社会更加和谐稳定。

　　（一）坚持创新驱动理念，着力建设智慧辽阳

　　加快经济结构战略性调整，推动信息化与一二三产业深度融合，催生经济增长新业态，大力培育和发展智慧经济。做优第一产业，高标准建成国家现代农业示范区。做强第二产业，以《中国制造2025辽阳行动纲要》为统领，改造提升传统产业，培育壮大战略性新兴产业，促进工业结构优化升级。做大第三产业，以集聚区建设为载体，大力发展特色服务业；以老城区改建为契机，提升传统服务业档次水平；以经济结构调整为主线，加快发展生产性服务业；以便民服务扩大消费为抓手，创新发展生活性服务业。

　　推进体制机制创新。主动融入沈阳经济区，打造“沈辽鞍营振兴产业带”。加快转变政府职能，持续推进简政放权，建成法治政府。深化市属国有企业改革。引导民营企业做大做强。深化投融资体制改革，推进政府和社会资本合作。创新对外开放体制机制，主动融入国家“一带一路”发展战略。

　　加快科技创新步伐。推动产业创新、企业自主创新和协同创新，把高新区和经济开发区建设成为创新型、智慧型园区。着力培养一线创新、专业技术和技能型人才，吸引更多人才到辽阳创新创业。推进信息技术广泛应用，加快建设智慧城市。

　　（二）坚持文化引领理念，着力建设文明辽阳

　　争创全国文明城市。践行社会主义核心价值观，着力构建廉洁高效的政务环境、民主公正的法制环境、公平诚信的市场环境、健康向上的人文环境、有利于青少年健康成长的社会文化环境、舒适便利的生活环境、安全稳定的社会环境、可持续发展的生态环境，全面提高广大市民的文明素质和生活品质。

　　建成国家历史文化名城。打造东北第一城品牌。加大历史文化遗产保护力度，做好汉魏壁画墓群等重点文物保护工作，完善和实施《历史文化名城保护规划》。建设历史文化街区，打造燕州城遗址公园、首山人文生态风景区，适时开发东京城，发掘明城墙等遗址。建设非物质文化遗产展示馆等基础设施，传承保护传统工艺和老字号。

　　推动文化事业和文化产业繁荣发展。文化强市建设进入省先进行列。实施文化“名牌、名品、名人”工程，扶持优秀文化产品创作生产。把文化旅游业培育成为支柱性产业。推进公共文化设施建设并免费向社会开放。建设市县博物馆、图书馆、文化馆及乡文化站等设施，实现“一村一室一广场”全覆盖。

　　（三）坚持绿色发展理念，着力建设美丽辽阳

　　改善生态环境。以建设国家级生态文明市为目标，深入实施“青山、碧水、蓝天、沃土”工程，打造生态宜居城市，到2019年，基本消灭重度雾霾天气；到2020年，城市空气环境质量优良天数的比例达到75%。

　　推进新型城镇化。提高城市建设水平，强化中心城区带动作用，引领新型城镇化发展方向。充分发挥河东新城的龙头作用，建设森林化滨水型城市。完善提升太子河历史文化景观带，有序开发太子岛。实施东辽阳老工业区搬迁改造工程，打造环境优美的宜居区。加强县城市政基础设施和公共服务设施建设，改善人居环境，增强集聚功能。加快重点镇建设，培育一批市级特色小城镇。深化户籍制度改革，实现存量农业转移人口市民化7.4万人。全面完成宜居达标村建设，80%的乡（镇）、村达到国家级标准。建设主体功能区，统筹推进集聚开发、分类保护和综合整治。

　　（四）坚持共享发展理念，着力建设幸福辽阳

　　坚持民生优先。每年都集中精力办好一批民生实事。深入推进就业工作，城镇就业年均实现3万人以上，城镇登记失业率控制在3.5%以内。建立健全覆盖城乡、更加公平、可持续的社会保障体系。实施精准扶贫，确保26492名贫困人口全部脱贫。

　　加强社会建设。办好人民满意教育，深入实施素质教育，实现学有所教。加强幼儿教育，公办园和普惠性民办园在园幼儿占65%以上。深化医药卫生体制改革，实行医疗、医保、医药联动和分级诊疗，确保病有所医。落实全面两孩政策。推进群众体育、竞技体育和体育产业协调发展。加强和创新社会治理，健全社会治安防控体系，认真解决群众合理诉求，强化安全生产管理，保障人民群众安居乐业。

　　完善城市功能。加强交通、能源、通信、地下管廊等城市基础设施建设，提高承载能力。加快发展智能交通和绿色交通，构建立体化交通运输体系。完善交通空间布局，建设四通八达的公路网络。大力发展公共交通，提高城乡公交覆盖率。加快推进城市公共设施、文化设施和商业设施建设。加强城乡住房建设，消除城市中心棚户区，加大城中村和城乡危房改造力度，实施老旧小区改造工程。推进城市管理智能化、规范化。

　　2016年是“十三五”的开局之年，做好今年的工作意义重大。我们要全面落实市委十一届十次全会决策部署，深入贯彻中央和省、市经济工作会议精神，坚持稳中求进工作总基调，狠抓“稳增长、调结构、促改革、惠民生、防风险”各项工作措施落实，着力加强结构性改革，在适度扩大总需求的同时，去产能、去库存、去杠杆、降成本、补短板，提高供给体系质量和效率，提高有效供给和有效需求的层次和水平，推进投资、消费、出口协同发力，三次产业协调发展，形成需求侧和供给侧支撑合力，确保实现“十三五”良好开局。

　　全市经济发展的主要预期目标是：地区生产总值增长6%左右，一般公共预算收入增长3%左右，固定资产投资增长8%左右，城乡常住居民人均可支配收入分别增长7%和7.5%左右。

　　为实现上述目标，我们要突出抓好以下九项工作：

　　（一）发挥投资、消费、出口的协调拉动作用，保持经济平稳增长

　　稳增长事关“十三五”良好开局和全面实现建成小康社会目标，仍然是今年全市经济工作的首要任务。

　　科学把握投资方向。切实发挥有效投资拉动增长的关键作用，以促项目开工撬动有效投资，以促项目达产助推经济增长。落实积极的财政政策并加力增效，广泛采用政府和社会资本合作方式，增加公共产品和公共服务的有效供给。落实金融扶持政策，鼓励企业技术进步，支持企业技术改造和设备更新，加快发展新兴产业。落实鼓励民间投资和市场准入的各项政策。严格控制落后产能，稳妥化解过剩产能。对230个投资亿元以上项目，落实领导包扶和定期调度制度，力促早开工、早达产。

　　促进消费潜力释放。充分发挥消费对稳增长的基础作用，扩大消费需求对经济增长的贡献。深入推进六大领域消费，加快形成服务消费、时尚消费、品质消费、农村消费等新的增长点。着力促进住房消费，坚持“三化”安置，落实好农民工市民化、公租房扩大到非户籍人口等政策，扩刚需、去库存，办好房交会，推动房地产业健康平稳发展。加快建设特色街区，积极发展“夜经济”，办好各类展销会。

　　提升外贸出口水平。有效发挥出口对稳增长的支撑作用，加快对外贸易转型升级。用好用足出口鼓励政策，建立外贸发展扶持资金，培育壮大出口基地。灵活运用代理出口方式，积极为企业出口创造条件。建立专业跨境电子商务平台，培育市场采购贸易，引导皮装裘皮等企业走出国门参与国际竞争。主动融入国家“一带一路”倡议，积极参与中蒙俄等国际经济合作走廊建设，加快“走出去”步伐。

　　着力抓好稳工业、去产能、降成本。强化经济运行监测，支持企业稳定生产。大力推进钢铁、水泥、矿产行业去产能，调整优化存量。开展降低企业成本专项行动，实施消费品质量提升工程，促进优质产品本地化配套。深入开展“千名领导干部包扶千户企业”活动，及时帮助企业解决实际困难。

　　（二）深入实施《中国制造2025辽阳行动纲要》，加快调整工业结构

　　把提升制造业水平作为工业经济的核心任务，设立专项推进基金，出台鼓励政策，力促制造业发展取得明显成效。

　　培育壮大智能制造业。抓好试点示范及推广应用工作，建设自动化、数字化车间，组织企业实施“机器换人”计划。发展高端装备和智能产品，推进无人机、数控机床、智能流量调节阀等实现产业化。大力支持电子信息产业园建设。拓展“互联网+”业务，加速“两化”深度融合。全年确保智能制造主营销售收入占全市规模以上制造业比重提高5个百分点。

　　加快工业转型升级步伐。以俄油加工优化增效改造、200万吨铝板带箔等重点项目建设为突破口，做大做强芳烃及精细化工、铝合金精深加工、装备制造及汽车零部件三个优势产业。推动钢铁、水泥企业向装配式建筑领域发展，力争钢铁转型升级项目投产达效。引导要素资源流向先进装备制造、新材料等战略性新兴产业，形成新的发展动能。

　　提高创新驱动能力。扩大政产学研合作成果，培育百户高成长型中小企业和科技创新百强企业，提升芳烃及精细化工等国家级企业技术中心研发创新能力，推进忠旺集团航空航天铝合金联合研发实验室建设。支持企业开展并购和上市融资。培训提升本地人才。设立引才基金，引进高端人才。加快建设中小微企业创业孵化基地和服务平台，推动大众创业、万众创新。推进产业战略联盟和行业协会建设。

　　（三）以建设国家现代农业示范区为统领，大力推进农业现代化

　　坚持用工业理念发展农业，加快转变农业发展方式。加速建设国家现代农业示范区，新建优质粮食、水果采摘和休闲农业3个示范区（带），新发展10个现代农业示范园。

　　着力构建现代农业生产供给体系。做优高效、特色和有机农业，加快建设优质粮食示范区、生产基地，确保粮食产量稳定在100万吨左右。推进蔬菜示范园和生产基地建设，积极发展现代设施农业小区。加快发展特色林果业，大力创建国家和省级水果示范园。提升现代农业保障水平，建设农技推广示范基地，引进推广新品种、新技术。推进畜牧业标准化、产业化发展，抓好动植物疫病防控工作。创建农业机械化示范区，提升耕种收机械化水平。完善农产品质量安全追溯体系。提高防灾减灾气象保障能力。

　　完善农业农村基础设施，努力补齐短板。统筹建设高标准农田，抓好列入省示范试点的万亩水稻种植区建设。实施一批骨干水利工程，完成灯塔灌区和浑沙灌区年度改造任务。加大农村安全水源建设力度，再解决一批农村人口的饮水安全问题。

　　深化农业农村改革创新。抓好省农村土地承包经营权确权登记“整市推进”试点工作，积极引导土地向新型农业经营主体流转，发展多种形式的适度规模经营。加强农业农村金融平台建设，扩大“种粮乐”、“政银保”担保额度和覆盖面。以新型农业经营主体为重点，拓展农业保险覆盖范围。

　　打好精准脱贫攻坚战。按照精准扶贫、精准脱贫要求，健全扶贫开发工作机制，因户施策、因人施策，因地制宜地实施发展生产脱贫、生态补偿脱贫、发展教育脱贫、社会保障兜底脱贫工程，年内确保1万人精准脱贫。

　　（四）突出产业特色，促进服务业大发展、快发展

　　深入实施服务业发展四年行动计划，推动生产性服务业专业化、集聚化，生活性服务业便利化、品质化。

　　加快服务业集聚区建设。合理布局白塔中央商贸区商业业态，加速推进万嘉国际生活广场等项目建设。建设站西专业市场集群和农产品流通区域中心，开工建设火车站西站房和站西广场。加速打造芳烃等化工产品研发集聚区。（上接2版）积极推进海宁皮革城跨境电子商务落户佟二堡。加快建设养老养生、水上娱乐、冰雪运动等项目，进一步丰富温泉旅游集聚区业态。

　　大力发展生产性服务业。积极引导工业企业分立生产性服务业，加速发展工业物流和研发设计业。推进现代综合物流园区建设。着力抓好农产品现代流通体系试点建设，形成完善的农产品直销网络。加快发展城市和农村电子商务。大力发展金融业，优化地方股份制银行股权结构，组建辽阳农商行、灯塔农商行，积极引进外埠金融机构。

　　培育壮大生活性服务业。落实文化、体育、旅游等十项发展生活性服务业重点任务。多措并举发展健康养老和家政服务业。打造特色旅游精品，支持弓长岭区争创国家生态旅游示范区，佟二堡争创国家4A级景区。开发燕州城等历史文化资源及东部山区自然生态资源，启动东京城、太子岛开发建设。

　　（五）加强城市建设管理，加速新型城镇化进程

　　全面落实我市《推进新型城镇化工作实施意见》。不断完善河东新城服务功能和基础设施，打造便利宜居环境，加速聚集人气。加强灯塔新城基础设施建设，提高承载能力。加快建设马伊新区和首山公园新区，优化县城环境。提升汤河新城城市设计水平，打造特色鲜明的风情小镇。抓好省建制镇试点工作，完善佟二堡新城功能。

　　加快老城区改建步伐。实施棚改项目34个，惠及居民1.1万户。对20个老旧小区进行综合改造。维修“弃管楼”286栋。新建韩国蔚山园、南环街街边游园，改造博爱园，启动青年湖公园改造工程。提高城市绿化美化亮化水平。

　　加强城乡基础设施建设。开工建设站前大型人防交通枢纽。开工建设北环路，开展西环路建设前期工作，积极进行南环路、东环路和沈辽大道建设可行性研究。启动前进街、爱民街连通铁西工程。实施新华路、文圣路贯通北环路工程。维修街巷道路28条，大中修、改扩建各级公路500公里以上。加强供电、燃气、给排水等设施建设，启动城市第二热源和垃圾处理设施建设前期工作。

　　坚持建管并重，构建智慧城市管理格局。完成《城市总体规划》修改工作，树立规划权威，严格执行规划。加快数字指挥中心建设，深入推进网格化管理，提升城市智能化管理水平。因地制宜研究治堵之策，缓解道路交通压力。鼓励社会资本建设运营城市停车场，规范泊车秩序。开展对违法建筑、占道经商、废品收购等专项整治，改善城市形象。

　　（六）加大环境保护和治理力度，切实优化生态宜居环境

　　坚持绿色发展、可持续发展，大力保护生态环境，全力推进美丽辽阳建设，努力构建人与自然和谐发展新格局。

　　深入实施青山工程。落实《青山保护规划》，实行严格的林地保护制度。推进封山育林、荒山造林，严防小开荒反弹。

　　加快推进碧水工程。全面落实“水十条”，加大沿河排污企业的查处力度，推动灯塔市、宏伟区污水处理厂二期工程建设。加强饮用水源保护，划定生态保护红线。强化危险化学品、危废固废、重金属、放射源等安全监管。启动土壤综合治理工程。

　　巩固蓝天工程成果。认真贯彻落实“国十条”，加速淘汰城区内燃煤锅炉，强化建筑工地扬尘治理，更新清洁能源公交车40台，完善应急响应机制，打好三年治霾第一战，努力还市民一片蓝天。抓好环保违规建设项目清理整顿，释放存量新动力。

　　深入开展宜居乡村建设。以农村环境整治为重点，抓好宜居示范镇和示范村创建工作，建设美丽乡村示范村28个。

　　（七）完善体制机制，依靠改革开放增强发展动力

　　加快转变政府职能。协同推进简政放权、放管结合、优化服务，再取消和下放一批行政审批事项。加强政府工作部门权力清单和责任清单建设，研究制定负面清单。清理“红顶”中介服务事项，取消不合法不合理收费。

　　深化市属国有企业改革，实现工业领域国有股全部退出竞争性行业。稳妥推进集体企业改革，完成国有工业企业“三供一业”分离移交年度任务。加快投资体制改革，对企业投资项目继续简化审批要件。严格政府投资项目管理。完善公共资源交易平台，以管理创新促进资源配置高效透明。深化财税体制改革，扩大“营改增”覆盖范围，完善政府债务风险管理制度。深化公立医院改革，完成两县（市）中医院综合改革，扎实推进分级诊疗工作。完成公车改革。进一步深化商事制度改革。

　　提升对外开放水平。突出产业链招商，积极引进一批牵动作用强的项目。坚持外资和内资并重，改进招商方式，实现利用外资总量、质量新突破。构建智慧政务公共服务体系，营造亲商、安商、富商的良好环境。加快打造“沈辽鞍营振兴产业带”，协同推进区域一体化发展。

　　（八）切实加强社会建设，让发展成果更好造福于民

　　巩固就业再就业工作成果。推进创业型城市建设，新增城镇实名制就业3万人，城镇登记失业率控制在3%以内，保持零就业家庭动态为零。

　　完善社会保障体系。实施机关事业单位养老保险制度改革。稳步扩大社保覆盖面，新增参保人员2.1万人。探索建立养老保险风险金机制，确保养老金及时足额发放。落实降低工伤、生育保险费率政策，推进建筑企业参加工伤保险“同舟计划”。加强社会救助能力建设，逐步提高城乡低保标准。落实残疾人保障政策。筹集公租房600套，改造农村危房813户。

　　推进各级各类教育均衡发展。加强义务教育发展基本均衡县（市）区建设，扩大优质公办幼儿园和普惠性民办幼儿园覆盖率，实施一般性普通高中提升工程。加快发展现代职业教育，推进校企合作。实施义务教育薄弱学校改造工程，完成四高中、二十五中整体改造。加强校园安全管理，确保校车安全。

　　提高公共卫生保障能力。加强医疗卫生服务体系和服务能力建设，强化医疗质量和技术管理，做好重大疾病防治工作，实施一批公共卫生防治项目。新建市九院门诊病房楼，建成市卫生信息中心、中心医院新城医院和辽阳康复医院。

　　积极发展文化体育事业。加速创建国家历史文化名城。建设“一卡通”社区图书馆。加大辽瓷资源开发力度，打造冮官屯陶瓷文化品牌。合理有效利用文化体育场所，丰富群众文体生活。提升竞技体育水平。

　　加强和创新社会治理。创新完善立体化社会治安防控体系，推进打防管控一体化、信息资源一体化、网上网下一体化，切实增强防控体系合力。坚持依法严打方针，严厉打击严重刑事犯罪、多发性侵财犯罪和网络犯罪，整治“黑拐枪、黄赌毒”等治安顽疾。加强信访工作，推进依法逐级走访和网上信访，有效解决各类社会矛盾。强化生产、消防、交通、食品药品等安全监管，提高突发事件应急处置和防灾减灾能力。支持发展慈善事业，动员社会力量参与解困济难。加强全民国防教育和国防动员建设，开展新一轮双拥模范城创建工作。加速创建全国文明城市。

　　（九）强化政府自身建设，努力提高法治能力

　　主动接受市人大及其常委会的法律监督和工作监督，及时向市人大及其常委会报告工作，认真执行市人大及其常委会的决议、决定和审议意见，认真办理人大代表的议案、建议。主动提请市人大常委会审议立法计划项目，积极配合做好立法工作。自觉接受市政协的民主监督，积极支持政协参政议政，提高政协提案办理质量。充分听取政协以及各民主党派、工商联、无党派人士、人民团体的意见和建议。

　　增强法治观念，不断提高依法行政能力。严格按照法定权限和法定程序行使权力、履行职责，加快建设职能科学、权责法定、执法严明、公开公正、廉洁高效、守法诚信的法治政府。强化对行政权力的制约监督，规范行政自由裁量权和涉企行政执法行为，完善纠错问责机制。

　　增强效能观念，不断提高政府执行能力。大力弘扬雷厉风行、真抓实干的工作作风。大兴调研之风，下基层、解难题、办实事。坚持治庸治懒治散，强化绩效考核，提高工作效率。

　　增强群众观念，不断提高为民服务能力。巩固“三严三实”专题教育成果，树牢执政为民的理念。强化服务意识，提高公共服务效率。树立诚信形象，提升政府公信力。全面推进政务公开，保障群众知情权和监督权。

　　增强廉政观念，不断提高拒腐防变能力。认真执行中央八项规定和省市委十项规定，严格落实党风廉政建设责任制，遵守并执行《准则》和《条例》。加强行政监察、审计监督、工作督查，把纪律挺在前沿，树立清正廉洁的政府形象。

　　各位代表，我们要树牢共享发展理念，牢记发展为了人民、发展依靠人民、发展成果由人民共享的使命，在全面做好各项民生工作的基础上，集中力量办好群众最期盼、受益最直接的20件民生实事，倾情倾力为民多谋福祉。

　　各位代表，今天的辽阳已经站在新的历史起点上，正朝着全面振兴的目标迈进。让我们在市委的坚强领导下，抢抓机遇，开拓进取，为全面建成小康社会，加快建设智慧文明美丽幸福新辽阳而努力奋斗！
